# Fostering sustainable investments through micro-investing platforms

**DOI:** 10.1038/s41598-023-48452-3

**Published:** 2023-12-01

**Authors:** Claudia Gonzalez-Arcos, Cristyn Meath, Peter Popkowski Leszczyc, Ernan Haruvy, Jake An

**Affiliations:** 1 Universidad Adolfo Ibáñez, Santiago, Chile; 2 University of Queensland, Brisbane, Australia; 3 McGill University, Montreal, Canada; 4 University of Technology Sydney, Ultimo, Australia

**Keywords:** Human behaviour, Climate-change mitigation, Environmental impact, Climate-change impacts, Climate-change mitigation, Environmental impact, Psychology and behaviour, Sustainability

## Abstract

We uncover the underlying factors that influence perceived trade-offs between sustainability and financial returns and risks, and the resulting real-world investment behaviour of micro-investors. Given the direct-to-consumer nature of new age investment platforms, the context for our study is framed within a consumer-centric context. Through a survey and conjoint experiment (383 investors), and analysis of actual investment decisions (for 89,744 micro-investors), we show that individual motives—specifically sustainability values and feelings of empowerment—are key drivers for sustainable investments, influencing willingness to forgo financial returns and the duration of investment. We provide practical implications for fostering sustainable investments through micro-investing platforms.

## Introduction

Extant literature about capital flows in the context of sustainable investing has predominantly focused on the demand side of sustainability funding, namely institutions seeking financial support for sustainability efforts such as non-profits and social enterprises^1^. There is limited understanding of the supply side of capital, particularly regarding individual investors (i.e., non-professional retail investors). Most discussions have focused on the role of organizations (e.g., corporate social responsibility), experienced private investors^2^, and institutional investors^3–5^. This study aims to bridge this gap in the literature by adopting a perspective from the supply side of capital for social impact. In this research, we aim to uncover the underlying factors that influence the perceived trade-offs between sustainability and financial returns and risks, and the resulting real-world behavioural outcomes in the context of micro-investors. Micro-investors are defined as individual investors, a specific subset of retail investors, who regularly invest small amounts through a micro-investing mobile-based platform^6^. Furthermore, given the direct-to-consumer nature of micro-investing platforms, and the context for our study, we take a consumer perspective.

Much of the investment capital originates from large investors, primarily institutional investors. Here we investigate the behaviour and impact of individual investors (general and micro-investors) with a focus on micro-investors. For the purposes of this study, we define general investors as non-professional individual investors ‘who buy and sell securities or funds (i.e. non-institutional investors)’^7^ and engage in investments through the use of a stockbroker or an online investing account.

Micro-investing platforms fall under the umbrella of democratized investing. This category includes micro-investing^8^ as well as peer-to-peer (P2P) lending^9^, micro-financing^10^ and crowdfunding^11,12^. These emerging platforms provide a wider portion of the population access to mainstream financial resources (e.g., financially vulnerable consumers^13^), thereby enabling investors to have a voice in capital allocation. Micro-investing platforms are designed to make investing accessible for individuals across all income levels^14^. This active involvement influences the course of capital flow, circumventing traditional barriers^9^. These emerging platforms, constituting a global micro-investment market of $1.8 trillion^15^, hold a crucial role in directing capital towards sustainable companies^16^.

Addressing critical sustainability challenges^17,18^ involves innovative approaches, such as micro-investing platforms to channel capital towards sustainable endeavors^19^. Consequently, this paper aims to determine how micro-investing platforms can foster sustainable investments. Achieving this goal involves tackling two pivotal challenges. The first challenge (*Research Question 1)* is in measuring micro-investors’ preference for SI and identifying critical factors that influence these preferences. The second challenge (*Research Question 2)* is in verifying whether the measured preference for SI translates to differential SI behaviour over time.

To address Research Question 1, in Analysis 1 we elicit micro-investors' sustainable values^20^ and their preferences for SI relative to expected returns and risk tolerance. Specifically, our study employs a survey combined with a conjoint experiment to quantify the extent to which investors are willing to (1) accept lower financial returns and/or (2) take on greater risk to invest in SI. Next, we delve into the factors that impact these two trade-offs, including individual values, preferences for sustainable investing (SI), attributes of SI funds (conjoint attributes), and demographic characteristics. Our data collection encompasses both general investors and micro-investors, as defined earlier.

To address Research Question 2, in Analysis 2, we investigate whether the measured preference for SI translates into actual SI behaviour. To that end, we analyse real-world investing data from a micro-investing platform. This analysis aims to determine if micro-investors, who demonstrate a stronger inclination toward prioritizing sustainability over financial returns, indeed allocate their investments into the platform’s sustainable fund. More specifically, Analysis 2 examines whether the willingness to accept (1) lower financial returns to invest in SI and (2) accept greater risk to invest in SI (from Analysis 1) are related to sustainable choices (i.e., actual portfolio choices after 12 months).

Measuring investors’ preference for SI is difficult as extant research has shown that investors’ preference for SI is often tainted with dual, and sometimes conflicting, goals of greater financial returns^21^ and positive social change^3^. Given that duality, the challenge lies in discerning investors' psychological trade-offs between sustainability and financial returns. Recent research has shown that the extent to which investors care about social impact, which can be measured by their willingness to pay additional fees for sustainable investments, is driven by an emotional, rather than calculative valuation of impact^2^. Research also shows differences between “sustainability-oriented investors” and other investors in terms of weight on non-financial returns^22,23^, distinguishing between sustainable investors, who invest for pro-social reasons, and other investors who invest for financial gain. The socially sustainable investors were shown to have higher social preferences, levels of education and trust, and are more likely to be left-wing and risk-averse^23^. Yet, differences may also exist amongst sustainability-oriented investors in terms of how much they are willing to sacrifice their financial returns or risks for greater social impact. Such differences in investors’ perceived trade-offs make capturing investors’ preference for SI more difficult but important because, without clearly quantifying such trade-offs and understanding the critical factors that influence these trade-offs, attempts to foster greater SI may remain less than optimal.

To identify critical factors that influence micro-investors’ preference for SI, we examine how individual motives, demographics, and preferences for sustainable investments influence preference for sustainability weighted by (1) Investment Return and (2) Level of Risk. Evidence suggests that individual motives, especially, altruistic and egoistic values can influence investors' preference to invest sustainably^24^. However, the questions of which specific sustainable values (i.e., biospheric, altruistic, hedonic, and egoistic values) are associated with perceived trade-offs between sustainability and financial returns, and what the relative importance of these values is, remain unanswered. Our findings regarding the effects of sustainable values provide a meaningful theoretical contribution to the literature on consumer (or investor) values and their impact on pro-environmental actions^25,26^.

Moreover, we examine micro-investors’ attitudes toward SI in terms of how SI can make them feel a sense of warm-glow and empowerment, thereby, responding to calls in the literature to further explore the consequences of warm-glow on sustainable investments^2^. We integrate the concept of empowerment to understand how empowered investors feel to address sustainability issues by investing in SI^27^. Finally, we investigate the impact of SI fund characteristics, such as whether the SI fund consists of companies that do good for the environment, do good for society, or both, and whether the SI fund screens for companies based on best-in-class, positive impact or exclusion criteria. By examining micro-investors’ sustainable values, their attitudes toward SI, and the SI fund characteristics that may influence the perceived trade-offs between sustainability, financial returns, and/or risk, we shed light on strategies that can be used to target specific investors, shape their attitudes toward SI, and design SI fund in a manner that can foster greater SI.

Supplementary Table 1 provides an overview of the different analyses conducted in this paper.

## Results

### Analysis 1A

The output of conjoint analysis consists of the investors’ utility weights for each attribute, which is used to calculate the trade-offs investors are willing to make between SI and (1) financial returns, and (2) risk. We regress the key dependent variables, how much people are willing to sacrifice their financial returns/accept risk to invest in SI, on individual motives, conjoint attributes, demographics, and control for whether the respondent is from the micro-investing platform sample or the general investor sample (Supplementary Table 4). Results show that, as expected, individual motives including altruistic, biospheric, and hedonic values, have a positive effect on preference for sustainability, while egoistic values have a negative effect. Perceived empowerment also has a significant positive effect on preference for sustainability, while warm-glow is not significant. Mixed funds, but not environmental or social funds, lead to a greater preference for sustainability. Women are more willing to accept lower returns for the sake of sustainability. Age is negatively associated with the willingness to accept greater risk for the sake of sustainability. Predicted sustainability included to control for the self-selection induced endogeneity was not significant. The platform (micro-investing versus general investor) had a significant positive effect, which indicates that it’s important to control for the difference between the two samples.

### Analysis 1B

We use the data from the same sample micro-investors, to estimate a predictive model for the willingness to accept lower returns on SI. We regress this trade-off metric on demographics including age, gender, income, and investor characteristics such as sign-ins (i.e., the number of times the customer has signed into the account since joining the service), investment balance amount, amount of return on investment, and the previously held portfolio before switching into SI. The purpose of this model is to determine which variables predict investors’ trade-offs between SI preferences and returns. Previous literature has found that women tend to be more sustainable^28^ and has observed a negative relationship between risk aversion and green consumption^29^. Consistent with these findings, we observe that both women and less risk-averse investors—those who invest in moderately conservative and moderately aggressive portfolios—are more likely to accept lower returns for sustainable investments (see Supplementary Table 5).

### Analysis 2A

We analyse the actual portfolios of micro-investors to investigate whether the willingness to accept a lower return for SI is associated with a higher likelihood of persistent SI behaviour (i.e., do they invest in SI and do they continue to invest in SI?). To achieve this, we match investors’ willingness to accept lower returns and accept greater risk to invest in SI, obtained from the conjoint analysis, with data on their actual micro-investment portfolio (12 months after the conjoint experiment). We investigate whether investors still hold SI after 1 year (using a logistic regression), and how long they hold SI (using a Cox regression, i.e., a survival model). The logistic model (Supplementary Table 6) shows that the more people are willing to forgo financial returns in order to invest in SI, the more likely they remain investing in SI after 1 year. Similarly, the survival analysis (Supplementary Table 6) shows that the more people are willing to accept lower returns to invest in SI, the less likely they will switch out of SI and the longer they are likely to remain investing in SI. We do not find a significant effect for willingness to accept greater risk to invest in SI (therefore we do not include this variable in subsequent analyses).

### Analysis 2B

We report on two predictive models that are used to estimate willingness to accept lower returns for SI for the platform’s other 89,744 micro-investors who have invested in SI at least once. After predicting micro-investors’ willingness to accept lower returns for SI*,* we examined its relationship with whether or not they remain investing in SI after one year. As shown in Supplementary Table 7, we find that the predicted willingness to accept lower returns for SI increases the likelihood of holding SI after one year (based on the logistic regression) and reduces the likelihood of switching out of SI (based on the survival model). In other words, those who are predicted to have a greater willingness to accept lower returns for SI are less likely to switch out of SI once they invest in SI. These results are consistent for different models and different estimation methods. Supplementary Table 8 provides the descriptive statistics for the investors in analyses 1B and 2B.

## Discussion

Although the debate around the real impact of sustainable investments to foster and facilitate sustainable business practices still remains^30,31^, there is evidence that companies are increasingly being held accountable by shareholders for ESG performance, particularly by global institutional investing firms^32^. As a result, companies are responding to investor and consumer pressure^33^. The role of investors in pushing the sustainability agenda has also been highlighted by research^34^ which finds social entrepreneurial intentions are influenced by Perceptions of Social Support, including the ability ‘to attract investors for an organization that wants to solve social problems’ p.115. While our research doesn’t test these hypotheses, it does contribute to the growing acknowledgement of the need for evidence to understand micro-investors’ psychological factors^35^ and the role of individual motives^36^ in supporting sustainable investing. Furthermore, the rapid growth of micro-investment platforms^15^ highlights the potential of the combined impact of small investors to support a transition to sustainable businesses and economies.

We developed and tested an experimentally derived measure of micro-investors’ psychological trade-offs between SI and financial returns, as well as the antecedents and behavioural consequences. The study finds conclusive evidence that micro-investors value sustainable investing and are willing to forego some financial gain to do so, offering a promising avenue for shifting capital toward sustainable efforts. Micro-investing platforms create a new source of sustainable capital flows towards sustainability-oriented companies by democratising retail investing^14^. Our analysis also identifies the preference for micro-investors to favour impact investments as opposed to best in class. Research argues the importance of impact investing if we are to create more meaningful sustainability initiatives in companies^30,37,38^. In doing so, our research contributes not only to the literature on the role and importance of specific values to pro-environmental behaviour^25,26^, but it also contributes evidence of the associated real-world investment behaviour. Furthermore, the identification of different values as well as sustainability investment and return/risk preferences between individuals using micro-investment platforms compared to traditional retail investment options provides an important and timely addition to extant knowledge which previously did not distinguish such differences in the rapidly growing consumer group.

Importantly, these insights enable more effective targeting of specific investors who are more likely to have a greater preference for SI, and more effective portfolio design to foster greater SI among micro-investors, supporting the acceleration of the transition to sustainable economies. For example, micro-investment platforms should offer positive impact portfolios of both environmental and social impact companies and marketing strategies that emphasise the platforms’ alignment with perceived empowerment and sustainability motives.

### Limitation and future research

A limitation of the current research is that we compared general vs. micro-investors from different countries. As a result, there may be regional differences in demographics, and differences in individual motives (e.g., due to cultural differences). Future research should aim to conduct a more granular analysis within specific regions or countries to better understand how cultural, economic, and demographic factors influence sustainable micro-investors' decision-making processes.

Our research has considered the trade-offs that sustainable micro-investors make with respect to returns on investment. Future research should consider to what extent these sustainable investors differ in their willingness to accept a lower return and/or greater risk for the sake of SI.

Showing the link between willingness to accept a lower return and actual future investments in sustainable assets is an important step towards measuring impact. However, while research points to the influence of small investors, particularly through greater ‘noise’ due to the greater number of investors, future research is needed to measure the extent to which this investor group’s actions lead to increased sustainable practices within organisations.

At the individual investor level, more research is needed particularly to investigate the extent to which micro-investing enhances empowerment, and in turn, stimulates sustainable behaviour.

## Methods

### Analysis 1A

*Regression Analysis of Conjoint data and Survey data from samples of micro-investors and general investors*. Supplementary Information 1 (1. Data Collection: Survey and Conjoint Study) provides full details for Analysis 1.

The survey and conjoint experiment are conducted with two distinct sets of investors—micro-investors and general investors, obtaining responses from 383 investors who had previously invested in SI. General investors were obtained from the CloudResearch consumer panel. Micro-investors’ data were obtained from a major micro-investing platform that operates in Australia, Indonesia, and Malaysia. The platform is listed on the Australian Securities Exchange, and the total value of their funds exceeds AUD$1 billion. The platform boasts over 665,000 active customers who can invest in one of seven different diversified portfolios of exchange-traded funds at any point in time. Among them is a socially responsible portfolio, which we refer to as sustainable investment (SI). 72.7% of SI is allocated to socially responsible exchange-traded funds, and the remaining is allocated to the money market and government bonds. Other portfolios are non-SI portfolios, including a conservative, moderately conservative, moderate, moderately aggressive, and aggressive portfolio, as well as a portfolio that provides investors exposure to cryptocurrency. There is no additional fee for changing the portfolio. The micro-investing platform enables users to invest their spare change by rounding up their everyday purchases to the nearest dollar and automatically investing it in their portfolio. Customers can also make direct deposits into their investment accounts. This makes investing accessible to small investors with limited capital, with the minimum investment required to open an account set at AUD$5.70% of their customers fall between the age range of 18 to 35, and the average value of customers’ portfolios is over AUD$3000.

A survey link, with an invitation to participate in a survey, was emailed to approximately 6000 customers of the micro-investing platform. The email was sent to those who had invested in the sustainable portfolio at least once and had a balance of over $5AUD. Out of the 6000 customers, 307 completed the conjoint study, resulting in a 5% completion rate, which aligns with previous survey studies conducted by the company.

Similarly, we shared a survey link inviting participation in the survey with the CloudResearch consumer panel to gather responses from general investors. Their extensive online prime panel, comprising over 50 million participants, was employed for this purpose. Participants were screened based on their prior experience with sustainable investments.

A conjoint choice task is used to measure investor (revealed) preferences for sustainable investment. Conjoint analysis has been widely used in marketing and other areas, yet the majority of research in social sciences uses survey research based on stated preferences^35^. A major advantage of using a conjoint model is that it lets investors make trade-offs between different attributes (e.g., between the percentage of sustainable firms in a fund, and the rate of return), and can refer to preferences from these choices (trade-offs). This is opposed to rating scales where investors provide ratings for different attributes (stated preferences). In this research, we use stated preferences obtained from a survey with data from conjoint choice experiments to uncover revealed preferences. This is a powerful method when morality and social concerns affect decision making^39^ and provides us with the ability to match self-identified preferences and motives with revealed preferences through hypothetical choices^40^. Specifically, in addition to respondents’ demographics and past investment experience, we asked investors about their individual motives for sustainability including – empowerment^1^ (e.g. “My investments provide a way for me to shift capital towards the sustainability causes I want to support”), warm glow^41^ (e.g. “I feel happy about myself when I contribute to/purchase sustainable investments”), and values^20^ [biospheric (e.g. “it is important to protect the environment”), hedonic (e.g. “it is important to have fun”), egoistic (e.g. “it is important to have money and possessions”), and altruistic (e.g. “it is important to take care of those who are worse off”)]. Other survey items related to investors’ attitudes toward SI (warm-glow and empowerment), and their demographics can be found in Supplementary Information 1. We observe differences between the two samples. Micro-investors are less wealthy and younger than general investors (Supplementary Table 3). Furthermore, the two samples differed in relation to altruistic, biospheric and hedonic, and egoistic values, and empowerment as well as their attitudes toward SI.

For the conjoint analysis, we collected a random sample of 307 customers from the micro-investing platform. Note, that this number is larger than the number of survey respondents with sustainable investing experience in Supplementary Table 3 (n = 190), since this sample also includes respondents without previous experience with sustainable investments. Respondents completed a Conjoint choice experiment where they chose among different investment options described through five key attributes (Supplementary Fig. 1). Attributes included (1) the fund focus (either environmentally sustainable, socially sustainable, or a combination of both), (2) the percentage of assets that are sustainable, (3) the Rate of return, (4) Risk level, and (5) Fund screening (either positive impact, exclusion). The attributes and attribute levels are shown in Supplementary Table 2. We use a hierarchical Bayes estimation method to estimate the parameters for the conjoint model.

Next, we conduct two regression models—one with a preference for SI weighted by the investment return and another with a preference for SI weighted by the level of investment risk as the dependent variable (obtained from the conjoint analysis). These measure the willingness to accept lower returns and/or accept higher risk for SI. This is based on the theoretical foundation that investors trade-off their preferences for sustainability with the rate of return, and/or the risk of the investment (similar to the sharp ratio). Specifically, the preference for SI weighted by the investment return is measured by the utility weight (part worth) range for sustainability [range High (76–100%) to Low (0–25%)], divided by utility weight (part worth) range for the rate of return [range High (14%) to Low (2%)]. Similarly, the preference for SI weighted by risk is measured by the utility weight (part worth) range for sustainability [range High (76–100%) to Low (0–25%)], divided by utility weight (part worth) range for the risk [range High to Low].

The conjoint attributes, individual motives, and demographics, obtained from the survey, are included in the model as the explanatory variables, as well as a dummy variable to control for the two different populations of investors. We use a two-stage estimation procedure to control for self-selection-based endogeneity since investors who have previously invested in SI are different from those who have not^42^. The first stage consists of estimating a probit model where we first calculate predicted sustainability and in the second stage, we include this variable in the regression model (see Supplementary Table 4).

### Analysis 1B

We estimate two predictive models, with the willingness to accept lower returns for SI (obtained from the conjoint analysis) serving as the dependent variable, using a linear regression and a random forest machine learning model. We construct the random forest model, as an alternative model to predict the willingness to accept lower returns for SI, which is a widely used ensemble learning method that operates by constructing multiple decision trees, using the random Forest library in R (version 4.2.2). We used grid search to determine the optimal combination of hyperparameters including the maximum number of terminal nodes and the number of trees, which converged to be 70 and 400, respectively (see Supplementary Table 7). Since willingness to accept greater risks for SI was not significant in Analysis 2, we do not estimate this model. The independent variables comprise data collected from investors on the micro-investment platform, including age, income, gender, sign-ins (indicating how frequently investors have logged into the platform), investment balance, return on investment, and the type of portfolio held before investing in SI (see Supplementary Table 6).

### Analysis 2A

*Conjoint estimates used to predict actual investment of micro-investors*. We determine whether the willingness to accept (1) lower financial returns to invest in SI and (2) accept greater risk to invest in SI (from Analysis 1) are related to sustainable choices based on actual portfolio choices after 12 months. We estimate both a logistic regression model (with the dependent variable being an indicator of whether the investor still holds SI after 1 year) and a Cox regression (survival) model, where the dependent variable is the duration of time the investor remains in SI. Both models aim to measure the likelihood that an investor continues to invest in SI (see Supplementary Table 5).

### Analysis 2B

We estimate a logistic and Cox regression (survival) model, similar to analysis 2, to examine whether the measure of estimated willingness to accept a lower return for SI from Analysis 3 is associated with a higher likelihood of persistent SI behaviour for the 89,744 micro-investors who have invested in SI at least once.

### Supplementary Information

Supplementary Information.

## Data Availability

The data for this study and the analysis codes are available from the corresponding author upon request. Due to a non-disclosure agreement, the sample data from the micro-investment platform require authorisation by the company.

## References

[1] Phillips SD, Johnson B (2021). Inching to impact: The demand side of social impact investing. J. Bus. Ethics.

[2] Heeb F, Kölbel JF, Paetzold F, Zeisberger S (2023). Do Investors Care about Impact?. Rev. Financ. Stud..

[3] Riedl A, Smeets P (2017). Why do investors hold socially responsible mutual funds?. J. Financ..

[4] Barber BM, Morse A, Yasuda A (2021). Impact investing. J. Financ. Econ..

[5] Bauer R, Ruof T, Smeets P (2021). Get real! Individuals prefer more sustainable investments. Rev. Financ. Stud..

[6] FINRA. Investing, Starting Small-What Is Micro Investing? October 25 2018. https://www.finra.org/investors/insights/micro-investing (2018).

[7] WEF. The Future of Capital Markets: Democratization of Retail Investing, Insight Report August 2022. World Economic Forum. (2022).

[8] An J, Briley D, Danziger S, Levi S (2023). The impact of social investing on charitable donations. Manage. Sci..

[9] Caldieraro F, Zhang JZ, Cunha M, Shulman JD (2018). Strategic information transmission in peer-to-peer lending markets. J. Mark..

[10] Galak J, Small D, Stephen AT (2011). Microfinance decision making: A field study of prosocial lending. J. Mark. Res..

[11] Ahlers GK, Cumming D, Günther C, Schweizer D (2015). Signaling in equity crowdfunding. Entrep. Theory Pract..

[12] Hervé F, Manthé E, Sannajust A, Schwienbacher A (2019). Determinants of individual investment decisions in investment-based crowdfunding. J. Bus. Finance Account..

[13] Salisbury LC (2023). Beyond income: Dynamic consumer financial vulnerability. J. Mark.ting.

[14] Morningstar. Exploring the Rise of Micro-Investing: Making Investing Accessible to All. https://www.morningstar.com/news/pr-newswire/20230820io88501/exploring-the-rise-of-micro-investing-making-investing-accessible-to-all (2023).

[15] Vickovich, A. Banks eye $1.8trn micro-investing market (The Australian Financial Review, October 20, 2021).

[16] Gomez, B. How socially conscious young investors are putting their money where their ideals are. CNBC. Socially conscious investors put their money where their ideals https://www.cnbc.com/2019/02/13/influx-of-young-investors-can-revitalize-sri.htmlare (cnbc.com) (2019).

[17] Rockstrom J (2009). A safe operating space for humanity: identifying and quantifying planetary boundaries that must not be transgressed could help prevent human activities from causing unacceptable environmental change, argue Johan Rockstrom and colleagues. Nature.

[18] IPCC. Summary for Policymakers. In: Climate Change 2022: Mitigation of Climate Change. Contribution of Working Group III to the *Sixth Assessment Report of the Intergovernmental Panel on Climate Change.* (Cambridge University Press, Cambridge, UK and New York, NY, USA, 2022).

[19] Antoncic, M. Efficiently allocating capital to transition to a sustainable economy. In *Sustainability, Technology, and Finance* 211–233 (Routledge, 2022).

[20] Steg, L., & Nordlund, A. Theories to explain environmental behaviour. *Environmental Psychology: An Introduction*. 217–227 (2018).

[21] Hartzmark SM, Sussman AB (2019). Do investors value sustainability? A natural experiment examining ranking and fund flows. J. Finance.

[22] Hornuf L, Stenzhorn E, Vintis T (2021). Are sustainability-oriented investors different? Evidence from equity crowdfunding. J. Technol. Transf..

[23] Degryse, H., Di Giuli, A., Sekerci, N., & Stradi, F. Sustainable Investments: One for the Money, Two for the Show *(SSRN Scholarly Paper 4411343)* (2023). ER//www.fia.org/investors/insights/micro-investing

[24] Brodback D, Guenster N, Mezger D (2019). Altruism and egoism in investment decisions. Rev. Financ. Econ..

[25] Crompton, T., & Kasser, T. *Meeting Environmental Challenges: The Role of Human Identity* **29**. (Godalming, UK: WWF-UK, 2009).

[26] Maio GR (2010). Mental representations of social values. Advances in Experimental Social Psychology.

[27] Boley BB, McGehee NG (2014). Measuring empowerment: Developing and validating the resident empowerment through tourism scale (RETS). Tour. Manage..

[28] Zhao Z, Gong Y, Li Y, Zhang L, Sun Y (2021). Gender-related beliefs, norms, and the link with green consumption. Front. Psychol..

[29] Essiz O, Yurteri S, Mandrik C, Senyuz A (2023). Exploring the Value-Action gap in green consumption: Roles of risk aversion, subjective knowledge, and gender differences. J. Glob. Mark..

[30] Busch T, Bauer R, Orlitzky M (2016). Sustainable development and financial markets: Old paths and new avenues. Bus. Soc..

[31] Friede G, Busch T, Bassen A (2015). ESG and financial performance: Aggregated evidence from more than 2000 empirical studies. J. Sustain. Finance Invest..

[32] Eccles RG, Klimenko S (2019). The investor revolution. Harv. Bus. Rev..

[33] Unnikrishnan, S, Biggs, C., & Singh, N. Sustainability Matters Now More Than Ever for Consumer Companies. https://www.bcg.com/publications/2020/sustainability-matters-now-more-than-ever-for-consumer-companies (2020).

[34] Hockerts K (2017). Determinants of social entrepreneurial intentions. Entrep. Theory Pract..

[35] Schwartz D, Loewenstein G, Agüero-Gaete L (2020). Encouraging pro-environmental behaviour through green identity labelling. Nature Sustain..

[36] Nielsen KS, Brick C, Hofmann W, Joanes T, Lange F, Gwozdz W (2022). The motivation–impact gap in pro-environmental clothing consumption. Nature Sustain..

[37] Unruh, G., *et al*. Investing for a sustainable future: Investors care more about sustainability than many executives believe. *MIT Sloan Manage. Rev.* **57**(4) (2016).

[38] Busch T (2021). Impact investments: A call for (re) orientation. SN Bus. Econ..

[39] Awad E (2018). The moral machine experiment. Nature.

[40] Louviere JJ, Hensher DA, Swait JD (2000). Stated Choice Methods: Analysis and Applications.

[41] Haruvy E (2020). Fundraising design: Key issues, unifying framework, and open puzzles. Mark. Lett..

[42] Heckman JJ (1976). The common structure of statistical models of truncation, sample selection and limited dependent variables and a simple estimator for such models. Ann. Econ. Soc. Meas..

