# Supplementary material for: Fostering sustainable investments through micro-investing platforms
Source: Sci Rep. 2023 Dec 1;13:21194. doi: 10.1038/s41598-023-48452-3 (PMC10692169; doi:10.1038/s41598-023-48452-3)
Supplement: Supplementary file 1 — Supplementary Information. [file 41598_2023_48452_MOESM1_ESM.docx]

**Supplementary Information**

**Fostering sustainable investments through micro-investing platforms**

Claudia Gonzalez-Arcos (Universidad Adolfo Ibáñez, Chile; University of Queensland, Australia)

*Cristyn Meath (University of Queensland, Australia)

Peter Popkowski Leszczyc (University of Queensland, Australia)

Ernan Haruvy (McGill University, Canada)

Jake An (University of Technology Sydney, Australia)

*Corresponding Author

**1. Data Collection: Survey and Conjoint Study**

Respondents first filled out a survey, after which they were redirected to another website (sawtooth software page) where they completed the conjoint experiment.

***1.1. Survey Questions***

**Screening questions:**

[Investment experience] Have you ever invested money in shares, bonds or managed funds?

Yes | No

[Sustainable investment experience] Have you ever invested money in one or more assets that you consider to be sustainable investments?

Yes | No

[Sufficient Knowledge] Is your investment knowledge sufficient to answer questions about investment allocations among different financial alternatives?

Yes | No

**Demographic variables:**

[Gender] What is your gender?

Male | Female | Other **|** Prefer not to say

[Age] What is your age?

18-25 | 26-35 | 36-45 | 46-55 | 55-65 | 65+

[Income] What is your household income?

*Participants choose among 9 income brackets*

[Education] What is your level of education?

Less than High School | high school or equivalent | post-high-school education other than university degree | Bachelor’s degree or equivalent | Postgraduate education

**Individual motives:**

| **Sustainable Values** (Steg et al. 2014) |  |
| --- | --- |
| Please rate the following statements, where 1 = not like me at all to 7 = very much like me.  **Biospheric Values** (1) It is important to [him/her] to prevent environmental pollution.  (2) It is important to [him/her] to protect the environment.  (3) It is important to [him/her] to respect nature. (4) It is important to [him/her] to be in unity with nature.  **Altruistic Values**  (1) It is important to [him/her] that every person has equal opportunities.  (2) It is important to [him/her] to take care of those who are worse off. (3) It is important to [him/her] that every person is treated justly. (4) It is important to [him/her] that there is no war or conflict. (5) It is important to [him/her] to be helpful to others.  **Hedonic Values**  (1) It is important to [him/her] to have fun.  (2) It is important to [him/her] to enjoy the life’s pleasures.  (3) It is important to [him/her] to do things [he/she] enjoys. **Egoistic Values**  (1) It is important to [him/her] to have control over others’ actions.  (2) It is important to [him/her] to have authority over others. (3) It is important to [him/her] to be influential. (4) It is important to [him/her] to have money and possessions. (5) It is important to [him/her] to work hard and be ambitious. | α =.916    α =.873  α =.914  α =.761 |

Please rate the following statements on a 7-point scale, where 1 = highly disagree to 7 = highly agree.

**Warm Glow** (Haruvy et al. 2020) α =.850

(1) I feel like I am a good person when I contribute to/purchase sustainable investments.

(2) I feel generous when I contribute to/purchase sustainable investments.

(3) I feel happy about myself when I contribute to/purchase sustainable investments.

**Empowerment** (Boley et al. 2014) α =.771

(1) Sustainable investing makes me feel more connected to other people trying to address
 sustainability issues.

(2) Addressing sustainability issues fosters a sense of community.

(3) I often discuss my sustainable investments with others.

(4) My investments provide a way for me to shift capital towards the sustainability causes I want to support.

Please wait, you will be redirected to a new website.

***1.2. Conjoint Experiment Choice Tasks and Design***

After the respondents landed on the website that hosted the conjoint study, they read the following text, and were asked to complete 15 choice tasks. We used a randomized design such that subjects were randomly exposed to different attribute levels (shown in Supplementary Table 2).

Next, we will ask you to make a number of choices between different investment options (you have the choice between 3 different investment options or a choice to select none of the three). Trade-offs are between different types of sustainable funds (fund focus). The proportions vary from very low (0 – 25%) to very high (75% - 100%) proportions of sustainable assets. Further attributes are the screening of companies included in a fund, the average rate of return of the fund, and the level of risk.

If these were your only options, which would you choose? (1 of 15)

Example of one (out of 15) choice task


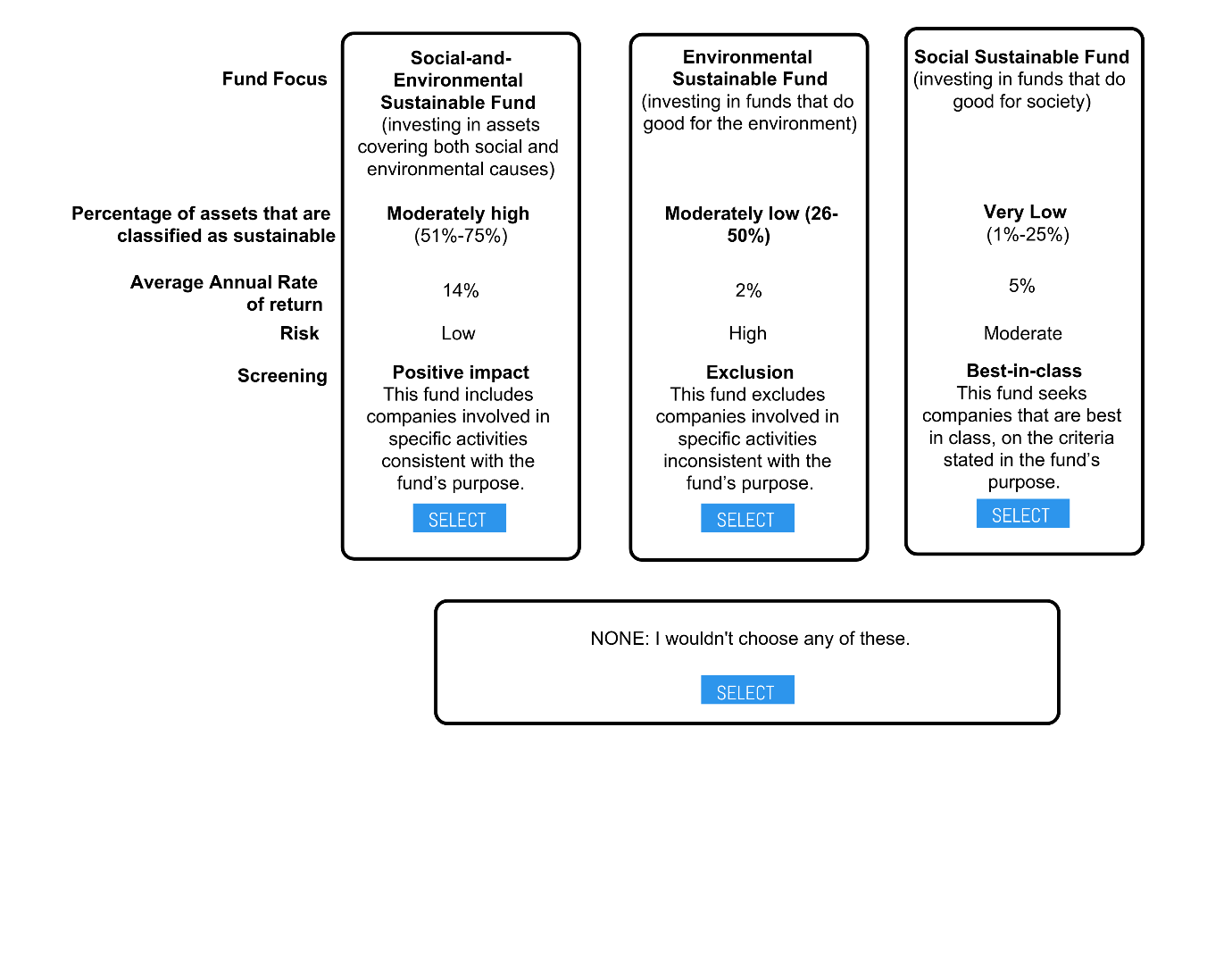


**Supplementary Figure 1. Example of conjoint experiment task**. Respondents had to choose among different hypothetical portfolio options. Each choice alternative was described on the basis of five attributes each with different levels (see Supplementary Figure 2). For each choice task, respondents selected their preferred portfolio option among three alternatives and a no-choice option; the attribute levels for the three choice options were (systematically) varied. Their choices allow us to measure their preference weights.

Final Screen

Thank you for participating in our study. The objective of this study was to study different trade-offs investors make related to the type and different attributes related to sustainable investments, level of risk, and the rate of return. Your payment will be directly deposited into your account at the completion of this study (please allow for several weeks for the amount to be processed).

1. **Supplementary Tables**

**Supplementary Table 1.** Overview and objectives of different analyses conducted in this paper

|  | **Overall research question: How can micro-investing platforms foster greater sustainable investment (SI)?** | | | |
| --- | --- | --- | --- | --- |
|  | **Analysis 1:** Measuring micro-investors’ preference for SI and identifying critical factors that influence these preferences. | | **Analysis 2:** Determine whether the measured preferences for SI translate to differential SI behaviour over time. | |
|  | **Analysis 1A** | **Analysis 1B** | **Analysis 2A** | **Analysis 2B** |
| **Research Question** | What factors influence investors’ perceived trade-offs between financial returns, risks and social impact? | Can investors’ preference for SI be predicted? | Do investors’ perceived trade-offs between financial returns, risks and social impact explain their actual SI choice after 12 months? | Can investors’ predicted preference for SI explain their actual SI portfolio choices after 12 months? |
| **Objective** | **First,** experimentally determine a measure of the degree to which investors are willing to accept (1) lower financial returns and/or (2) greater risk to invest in SI. **Second,** determine how these measures are influenced by individual motives, conjoint attributes, and demographic characteristics. | Estimate a predictive model for willingness to accept lower returns to invest in SI. | Determine whether the willingness to accept (1) lower financial returns to invest in SI and (2) accept greater risk to invest in SI (from Analysis 1) are related to sustainable choices (i.e., actual portfolio choices after 12 months). | Determine whether the willingness to accept lower financial returns to invest in SI (from analysis 3) is associated with a higher likelihood of persistent SI behaviour for the 89,744 micro-investors on the platform. |
| **Method** | Survey, conjoint experiment & regression analysis | Regression analysis | Logistic regression & survival analysis | Logistic regression & survival analysis |
| **Dependent variable** | Preference for sustainability weighted by the investment return. | Willingness to accept lower returns for SI. | Invest in Sustainable port-folio or not (Logistic model) after 12 months.  Time invested in sustainable portfolio (Cox model) during the 12 months. | Invest in Sustainable portfolio or not (Logistic model) after 12 months. Time invested in sustainable portfolio (Cox model) during the 12 months. |
| **Sample size** | 383 (193 General investors + 190 Micro-investors) | 307 Micro-investors | 307 Micro-investors | 89,744 Micro-investors |
| **Results** | Tables 3 & 4 | Table 5 & 8 | Table 6 | Table 7 & 8 |

**Supplementary Table 2.** Specific attributes and levels used in the conjoint experiment.^a^

| **Attributes** | **Attribute Levels** |
| --- | --- |
| Fund focus | **Social Sustainable Fund** (investing in funds that do good for society)  **Environmentally Sustainable Fund** (investing in funds that do good for the environment)  **Social-and-Environmentally Sustainable Fund** (investing in assets covering both social and environmental) |
| Percentage of assets that are classified as sustainable. | Low (0-25%) \| Moderately low (26-50%) \| Moderately high (51-75%) \| High (76-100%) |
| Average Rate of return | 2%, 5%, 8%, 11%, 14%, |
| Risk | Low \| Moderate \| High^b^ |
| Screening | **Positive impact** (this fund includes companies involved in specific activities consistent with the fund’s purpose)  **Exclusion** (this fund excludes companies involved in specific activities inconsistent with the fund’s purpose)  **Best-in-class** (this fund seeks companies that are best in class, on the criteria stated in the fund’s purpose) |

^a^In the 15 choice tasks respondents selected between three alternatives described based on these five attributes. Attribute levels were randomly selected for each respondent and each choice task.

^b^Risk is frequently defined on a scale from low or high, and which is similar to investment categories like conservative, moderate and aggressive (e.g., Wang, Keller and Siegrist, 2011).

**Supplementary reference:**

Wang, Mei, Carmen Keller, and Michael Siegrist. "The less You know, the more You are afraid of—A survey on risk perceptions of investment products." *Journal of Behavioral Finance* 12, no. 1 (2011): 9-19.

**Supplementary Table 3.** Average values for micro-investors and general investors

|  |  | | Micro-investors platform | | General Investors Panel | | T-test^a^ | P-value |
| --- | --- | --- | --- | --- | --- | --- | --- | --- |
| Category | Variable | Mean | | Std Error | Mean | Std Error |  |  |
| Demogra-phics | Female | 0.453 | | 0.036 | 0.332 | 0.034 | 2.440 | 0.015 |
|  | Age | 36.768 | | 0.885 | 38.705 | 0.872 | 1.560 | 0.120 |
|  | Income | $66,689 | | 3042 | $82,565 | 4054 | 2.240 | 0.026 |
| Individual  Motives | Altruistic values | 6.266 | | 0.050 | 5.684 | 0.090 | 5.790 | < 0.001 |
|  | Biospheric values | 6.287 | | 0.834 | 5.632 | 1.177 | 6.210 | < 0.001 |
|  | Hedonic values | 6.028 | | 0.846 | 5.652 | 1.154 | 3.560 | 0.001 |
|  | Egoistic values | 3.716 | | 0.067 | 3.992 | 0.088 | 2.510 | 0.013 |
|  | Empowerment | 4.957 | | 0.077 | 4.802 | 0.089 | 1.660 | 0.097 |
|  | Warm glow | 5.294 | | 0.077 | 5.339 | 0.082 | 0.400 | 0.690 |
| Preferences  for attributes of sustainable investments  funds (conjoint attributes) | Social fund | 0.058 | | 0.017 | 0.238 | 0.031 | 5.110 | < 0.001 |
|  | Environmental fund | 0.205 | | 0.029 | 0.394 | 0.035 | 4.100 | < 0.001 |
|  | Mixed fund | 0.739 | | 0.032 | 0.368 | 0.035 | 7.800 | < 0.001 |
|  | Best focus | 0.347 | | 0.035 | 0.544 | 0.036 | 3.940 | < 0.001 |
|  | Positive focus | 0.416 | | 0.036 | 0.264 | 0.032 | 3.160 | 0.002 |
|  | Exclusive focus | 0.237 | | 0.031 | 0.192 | 0.028 | 1.080 | 0.283 |
|  | Relative to risk tolerance | 1.125 | | 0.072 | 0.114 | 0.083 | 9.200 | < 0.001 |
|  | Relative to expected return | -0.061 | | 0.049 | -0.711 | 0.078 | 7.020 | < 0.001 |
|  | No. observations | 190 | |  | 193 |  |  |  |

^a^T-test for the difference in value for Micro-investors versus Cloud Research investors

N = 383

**Supplementary Table 4.** Regression estimates. Dependent variable: Preference for sustainability weighted by (1) Investment Return and (2) Level of Risk

|  |  | Investment Return ^a^ | | Level of Risk ^b^ | |
| --- | --- | --- | --- | --- | --- |
|  |  | coefficient | St. error | coefficient | St. error |
|  | Constant | 0.820 | 1.139 | 3.472* | 1.444 |
| Individual | Altruistic values | 0.169** | 0.053 | 0.175** | 0.067 |
| Motives | Biospheric values | 0.246** | 0.089 | 0.259* | 0.112 |
|  | Hedonic values | 0.178** | 0.051 | 0.144* | 0.064 |
|  | Egoistic values | -0.117* | 0.051 | -0.149* | 0.065 |
|  | Warm glow | 0.047 | 0.055 | -0.017 | 0.070 |
|  | Empowerment | 0.184** | 0.056 | 0.253** | 0.071 |
|  | Platform | -0.534** | 0.160 | -1.002** | 0.203 |
| Conjoint | Social Fund | -0.012 | 0.128 | 0.049 | 0.163 |
| Attributes | Mixed Fund | 0.269** | 0.100 | 0.267* | 0.127 |
|  | Best focus | 0.138 | 0.110 | -0.008 | 0.140 |
|  | Positive focus | 0.042 | 0.115 | 0.073 | 0.146 |
| Demographics | Female | 0.233* | 0.093 | 0.140 | 0.118 |
|  | Age | -0.003 | 0.005 | -0.012 | 0.007 |
|  | Income | -0.063 | 0.063 | 0.009 | 0.080 |
|  | Predicted Sustainability | -1.218 | 1.074 | -1.898 | 1.362 |
|  | R-square | 0.342 |  | 0.302 |  |

| N = 383; *p < 0.05; **p < 0.01; standard errors are in parentheses  ^a^ Willingness to accept lower returns for SI ^b^ Willingness to accept greater risk to invest in SI |
| --- |

#### **Supplementary Table 5.** Predictive Models of the Willingness to Accept Lower Returns

|  | Willingness to Accept Lower Returns for SI  (OLS) | Willingness to Accept Lower Returns for SI  (Random Forest) |
| --- | --- | --- |
| Intercept | 1.22*  (0.71) |  |
| Log (Age) | 0.09  (0.15) | 18.16 |
| Log (Income) | −0.02  (0.02) | 4.99 |
| Female | 0.15*  (0.08) | 3.85 |
| Log (Signin) | 0.03  (0.03) | 17.37 |
| Log (Balance) | −0.03**  (0.01) | 18.54 |
| Log (Returns) | −0.03  (0.07) | 23.57 |
| Previous Portfolio: Moderately Conservative | 0.49**  (0.20) | 16.95 |
| Previous Portfolio: Moderate | 0.15  (0.15) |  |
| Previous Portfolio: Moderately Aggressive | 0.22*  (0.14) |  |
| Previous Portfolio: Aggressive | −0.05  (0.12) |  |
| Previous Portfolio: NA | 0.12  (0.19) |  |
| Previous Portfolio: Bitcoin | −0.06  (0.24) |  |
| Previous Portfolio: Custom | −0.13  (0.53) |  |
| N = 307; *p < 0.05; **p < 0.01; standard errors are in parentheses; The numbers for the random forest predictive model indicate variable importance, whereby higher numbers indicate greater variable importance. | | |

#### **Supplementary Table 6.** Effect of the Willingness to Accept Lower Returns for SI on Holding Sustainable Investment After One Year

|  | Cox Model | Hazard Ratio | Logistic Model | Odds Ratio |
| --- | --- | --- | --- | --- |
| Intercept |  |  | −1.98  (2.55) | 0.14 |
| Willingness to Accept Lower Returns for SI | −0.78**  (0.38) | 0.46 | 0.85**  (0.42) | 2.35 |
| Willingness to Accept Greater Risks for SI | 0.04  (0.03) | 1.04 | −0.04  (0.03) | 0.96 |
| Log (Age) | −0.74  (0.68) | 0.48 | 0.60  (0.72) | 1.82 |
| Log (Income) | −0.11**  (0.05) | 0.89 | 0.14**  (0.06) | 1.15 |
| Female | −0.49  (0.37) | 1.63 | −0.60  (0.40) | 0.55 |
| Log (Signin) | −0.19  (0.15) | 0.83 | 0.07  (0.15) | 1.08 |
| N = 307; *p < 0.05; **p < 0.01; standard errors are in parentheses | | | | |

#### **Supplementary Table 7.** Effect of the Estimated Willingness to Accept Lower Returns on Holding Sustainable Investment After One Year

|  | Holding sustainable investments after one year  (Willingness to Accept Lower Returns for SI Estimated via OLS) | | Holding sustainable investments after one year  (Willingness to Accept Lower Returns Estimated for SI via Random Forest) | |
| --- | --- | --- | --- | --- |
|  | Cox Model | Logistic Model | Cox Model | Logistic Model |
| Intercept |  | −0.91**  (0.11) |  | −0.92**  (0.11) |
| Estimated Willingness to Accept Lower Returns for SI | −0.22**  (0.03) | 0.51**  (0.04) | −0.19**  (0.03) | 0.50**  (0.04) |
| Log (Age) | −0.23**  (0.02) | 0.18**  (0.03) | −0.23**  (0.02) | 0.17**  (0.03) |
| Log (Income) | −0.04**  (0.00) | 0.07**  (0.00) | −0.04**  (0.00) | 0.07**  (0.00) |
| Gender | 0.41**  (0.01) | −0.55**  (0.02) | 0.42**  (0.01) | −0.57**  (0.01) |
| Log (Signin) | 0.08**  (0.00) | −0.23**  (0.01) | 0.07**  (0.00) | −0.22**  (0.01) |
| N = 89,744; *p < 0.05; **p < 0.01; standard errors are in parentheses | | | | |

#### **Supplementary Table 8.** Descriptive Statistics for Consumer Samples in Analysis 3 and 4

|  | | Conjoint respondents | | Micro-investors who invested in SI at least once | |
| --- | --- | --- | --- | --- | --- |
| Variable | Mean | | Std Error | Mean | Std Error |
| Female | 0.48 | | 0.03 | 0.36 | 0.00 |
| Age | 37.50 | | 0.65 | 34.88 | 0.03 |
| Income | 49,512.40 | | 964.70 | 49,168.91 | 50.39 |
| Log (Signin) | 99.34 | | 14.53 | 49.97 | 0.65 |
| Willingness to Accept Lower Returns for SI | 1.08 | | 0.04 | NA | NA |
| Willingness to Accept Greater Risks for SI | 4.41 | | 0.35 | NA | NA |
| Estimated Willingness to Accept Lower Returns for SI (OLS) | NA | | NA | 1.02 | 0.00 |
| Estimated Willingness to Accept Lower Returns for SI (random forest) | NA | | NA | 1.07 | 0.00 |
| No. observations | 307 | |  | 89,744 |  |
